# Paper-Based Fluorescent Sensor for Rapid Multi-Channel Detection of Tetracycline Based on Graphene Quantum Dots Coated with Molecularly Imprinted Polymer

**DOI:** 10.3390/polym16172540

**Published:** 2024-09-08

**Authors:** Linzhe Wang, Jingfang Hu, Wensong Wei, Yu Song, Yansheng Li, Guowei Gao, Chunhui Zhang, Fangting Fu

**Affiliations:** 1 Beijing Key Laboratory of Sensor, Beijing Information Science & Technology University, Beijing 100101, Chinasongyu@bistu.edu.cn (Y.S.);; 2 Institute of Food Science and Technology, Chinese Academy of Agricultural Sciences/Key Laboratory of Agricultural Product Processing, Ministry of Agriculture, Beijing 100193, China; 3 Key Laboratory of Modern Measurement and Control Technology, Ministry of Education, Beijing Information Science and Technology University, Beijing 100192, China; 4 State Key Laboratories of Transducer Technology, Shanghai Institute of Microsystems and Information Technology, Chinese Academy of Sciences, Shanghai 200050, China; 5 Zibo Institute for Digital Agriculture and Rural Research, Zibo 255051, China

**Keywords:** graphene quantum dots, molecularly imprinted polymer, paper-based fluorescent sensor, tetracycline

## Abstract

In this paper, we developed a paper-based fluorescent sensor using functional composite materials composed of graphene quantum dots (GQDs) coated with molecularly imprinted polymers (MIPs) for the selective detection of tetracycline (TC) in water. GQDs, as eco-friendly fluorophores, were chemically grafted onto the surface of paper fibers. MIPs, serving as the recognition element, were then wrapped around the GQDs via precipitation polymerization using 3-aminopropyltriethoxysilane (APTES) as the functional monomer. Optimal parameters such as quantum dot concentration, grafting time, and elution time were examined to assess the sensor’s detection performance. The results revealed that the sensor exhibited a linear response to TC concentrations in the range of 1 to 40 µmol/L, with a limit of detection (LOD) of 0.87 µmol/L. When applied to spiked detection in actual water samples, recoveries ranged from 103.3% to 109.4%. Overall, this paper-based fluorescent sensor (MIPs@GQDs@PAD) shows great potential for portable, multi-channel, and rapid detection of TC in water samples in the future.

## 1. Introduction

Antibiotics are a class of compounds, either synthesized or naturally occurring, that play a critical role in treating bacterial infections and have been essential in agriculture, medical treatment, and the aquaculture industry. However, many antibiotics are not fully metabolized by humans or animals and are excreted in their active forms [1]. These active compounds enter waterways through sewage systems and agricultural runoff, disrupting aquatic ecosystems [2]. While antibiotics have revolutionized modern medicine since their discovery, their abuse has simultaneously led to a significant global crisis, primarily due to the rise of antibiotic-resistant bacteria. According to a report published in The Lancet, global antibiotic resistance directly caused approximately 1.27 million deaths in 2019 and is associated with nearly 4.95 million deaths [3].

Furthermore, tetracycline (TC) in water bodies can adversely affect microbial communities, which are essential for nutrient cycling and organic matter decomposition. This disruption can lead to decreased water quality, impacting aquatic organisms from plants to fish [4]. Moreover, continuous low-level exposure to TC through drinking water can lead to chronic health issues, including allergies, disruption of gut microbiota, urinary tract infections, and so on [5]. Current methods for TC testing include High-Performance Liquid Chromatography (HPLC) [1], Liquid Chromatography–Mass Spectrometry (MS) [6], and Enzyme-Linked Immunosorbent Assay (ELISA) [7]. However, these methods are limited by their long detection cycles, complex pretreatment processes, and expensive equipment. Therefore, the severity of this issue highlights the urgent need to develop a rapid, low-cost, and point-of-care testing (POCT) method for TC detection in water.

Paper-based analytical devices (PADs) are a novel POCT platform that has recently attracted increasing attention due to their cost-effectiveness and usability. Compared with traditional materials such as silicon, glass, and PDMS, it offers superior features such as low cost, portability, eco-friendliness, and biocompatibility, allowing for non-specialists’ rapid on-site testing [7]. Up to now, PADs have been integrated with various detection techniques such as colorimetry [8], electrochemistry [9], and fluorescence [10] to realize qualitative and quantitative measurements. These attributes have led to widespread applications in clinical diagnosis, food safety, and environmental monitoring, particularly in resource-limited areas.

Molecularly imprinted polymers (MIPs) are materials designed to mimic the recognition properties of biosystems, such as antibodies and enzymes. MIPs are created by polymerizing functional monomers around template molecules, which are then removed, leaving a three-dimensional network with cavities complementary to the template in size, shape, and functional groups [11]. MIPs can selectively identify and bind to target molecules and have advantages such as high selectivity, stability, low cost, and ease of manufacture, making them a reliable alternative to traditional analytical methods.

Fluorescence detection is a highly sensitive analytical technique used in various fields. The detection principle involves the fluorescence intensity enhancement or quenching caused by the direct or indirect interaction between the fluorophore and the target molecule. By detecting the change in the fluorescence intensity, the concentration of the target molecule can be determined. Various fluorescent materials, such as quantum dots [12], organic dyes [13], and metal nanoclusters [14], have been used to develop paper-based fluorescent sensors. Among them, quantum dots (QDs) have become a research hotspot because of their high fluorescence quantum yield, large Stokes shift, and stability [15]. Chen et al. [16] used a CdTe QDs-ZnTPyP composite and constructed a PAD for rapid caffeine detection. Qin et al.’s [17] modified cellulose paper with Mn-doped ZnS QDs was used to detect three heavy metal ions in water samples. However, heavy metal-based QDs are highly toxic and increase environmental risks. At the same time, graphene quantum dots (GQDs), due to their carbon structure and surface functional groups, are generally considered more eco-friendly and biocompatible. Therefore, GQDs are more suitable for applications in biomedical and environmental testing.

Herein, we developed a paper-based fluorescent sensor for the selective detection of TC in water. The fluorescence sensing mechanism was based on fluorescence quenching triggered by electron transfer between TC molecules and GQDs. To the best of our knowledge, it was first observed that GQDs coated with MIP as the sensing strategy on PADs were used to realize TC detection. This method transfers the liquid-phase GQD fluorescence detection system onto the PADs, improving the sensor’s portability and simplifying the testing procedure. Compared to conventional methods that measure only one sample at a time, this sensor can test up to 96 samples simultaneously by using a microplate reader, greatly improving multiplex detection capability while maintaining good sensitivity and selectivity. Our proposed MIPs@GQDs@PAD sensor demonstrated good analytical ability in resource-limited environments, opening up a new way for point-of-care testing of antibiotics.

## 2. Materials and Methods

### 2.1. Materials

Graphene quantum dots (GQDs) were purchased from XFNANO Materials Tech Co., Ltd. (Nanjing, China). The N-Hydroxysuccinimide (NHS), Tetramethoxysilane (TEOS), 1-(3-Dimethylaminopropyl)-3-Ethylcarbodiimide (EDC), (3-Aminopropyl)triethoxysilane (APTES), and 2-Morpholinoethanesulfonic Acid (MES) were purchased from Aladdin Chemistry Co., Ltd. (Shanghai, China). Acetic acid, hydrochloric acid (HCl), anhydrous ethanol, Ammonia (NH_3_·H_2_O, 25%), and ultra-dry methanol were purchased from Sinopharm Chemical Reagent Co., Ltd. (Shanghai, China). Tetracycline (TC), oxytetracycline (OTC), chloramphenicol (CAP), norfloxacin (NOR), and ofloxacin (OFX) were purchased from Macklin Biochemical Technology Co., Ltd. (Shanghai, China). Whatman No. 1 filter paper was purchased from GE company (Shanghai, China). All reagents were of analytical grade and used as received. Ultrapure water (18.2 MΩ) was used throughout this experiment.

### 2.2. Instruments

Fluorescence spectrums were conducted with a spectrophotometer (F97, Lengguang Technology, Shanghai, China). Fluorescence intensities were obtained from a microplate reader (infinite 200, Deacon, Shanghai, China). Fluorescence microscopy images were observed using a fluorescence microscope equipped with a CCD camera (ECLIPSE Ti-80i, Nikon, Tokyo, Japan). The morphology of the sensing surface was analyzed with a scanning electron microscope (Gemini 560, ZEISS, Oberkochen, Germany), operating in a vacuum mode. Fourier-transform infrared spectroscopy (FT-IR) was carried out with a Fourier-transform infrared spectrometer (Nicolet iS5, Thermo Fisher Scientific, Waltham, MA, USA).

### 2.3. Synthesis of GQDs@PAD

The PADs were designed by using drawing software (Adobe Illustrator 24.0.1), and a paper cutting machine (M1, XTOOL, Shenzhen, China) was used to cut Whatman No. 1 filter paper into 3.5 mm diameter circles. Subsequently, the PADs were immersed in a 0.1 mol/L HCl solution for 20 min and then rinsed three times with water to remove excess HCl. Afterward, the PADs were soaked in a 10 mL ethanol/water (50%) solution containing 80 µL of APTES and oscillated at 120 rpm for 3 h, then rinsed three times with water to remove the excess ethanol. A 6 mL solution containing 10 mg/mL EDC and 5 mg/mL NHS was freshly prepared using a 50 mM MES buffer (pH = 5.2), then 6 mg of GQDs was added and sonicated for 15 min. The paper was placed in the above solution and oscillated at 120 rpm for 3 h in the dark to allow for the GQDs to graft onto the paper substrate through covalent bonding. The obtained paper was denoted as GQDs@PAD.

### 2.4. Synthesis of MIPs@GQDs@PAD

The MIPs@GQDs@PAD was prepared according to a modification of the method reported in the literature [1]. Briefly, 80 µL of APTES as the functional monomer and 5 mg of TC as the template molecule were dispersed into 10 mL of ultrapure water and then oscillated at 130 rpm for 30 min, allowing APTES to pre-polymerize with TC molecules through self-assembly. Immediately after that, 80 µL of a crosslinker (TEOS) and 80 µL of a catalyst (NH_3_·H_2_O, 25%) were added. After about 30 min, when the white turbidity was observed at the bottom of the container, GQDs@PAD was added and oscillated for 12 h in the dark. In the final step, MIPs@GQDs@PAD was obtained using methanol/HCl (0.5 mol/L, 9:1, *v*/*v*) as the eluent to remove template molecules, followed by rinsing with water. The prepared MIPs@GQDs@PAD was stored in the dark at 4 °C. Furthermore, non-imprinted polymer PADs (NIPs@GQDs@PAD) were prepared as a control, following the same procedure except that no templates were added.

### 2.5. Fluorescence Analysis

Compared to previously reported paper-based fluorescence sensors, this MIPs@GQDs@PAD can be used with an enzyme microplate reader for more convenient and straightforward measurements. Firstly, the sensors were transferred to a 96-well black flat-bottomed microplate (Corning, Corning, NY, USA), and 10 µL of the TC water samples was added and allowed to absorb for 15 min. The fluorescence intensity of MIPs@GQDs@PAD was detected and recorded using a microplate reader with an excitation wavelength (λex) of 360 nm, a gain of 100, slit widths of 10 nm, and an emission wavelength (λem) range from 420 to 500 nm.

## 3. Results and Discussion

### 3.1. The Fabrication and Sensing Mechanism of MIPs@GQDs@PAD

**Figure 1:**
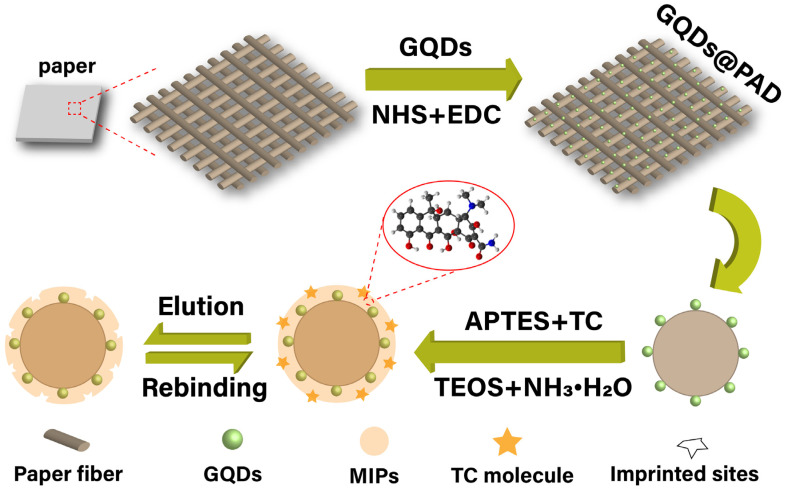
A schematic illustration of the fabrication process of the MIPs@GQDs@PAD.

The fabrication of this paper-based fluorescent sensor (MIPs@GQDs@PAD) and the sensing mechanism for TC detection are discussed. We chose chemical grafting to modify GQDs instead of physical deposition because GQDs modified on paper substrates through physical deposition can easily agglomerate, leading to fluorescence quenching [2]. GQDs and paper substrates connected through covalent bonding are more stable, and the GQD distribution is more uniform. Before grafting GQDs, the filter paper was soaked in HCl for 20 min to effectively remove impurities and activate the hydroxyl groups (-OH) on the paper surface, thus inducing the APTES and the hydroxyl groups to graft amino groups (-NH_2_) [3]. Subsequently, the amino groups connect with the carboxyl groups (-COOH) on the edges of the GQDs through an amidation reaction catalyzed by EDC and NHS, and GQDs are firmly grafted onto the paper substrate. Before the synthesis of MIPs, the amino groups of the APTES monomer interacted with the hydroxyl groups of the TC through electrostatic interaction (average bond length ≈ 1.70 Å) and with the carboxyl groups of the TC through hydrogen bonds (average bond length ≈ 2.30 Å), to form pre-polymerized complexes [4]. Afterward, APTES and TEOS were hydrolyzed to form silanol groups (Si-OH) catalyzed by NH_3_·H_2_O. The silanol groups then underwent condensation reactions to form siloxane bonds (Si-O-Si), forming a three-dimensional MIP network [5]. The fabrication of the MIPs@GQDs@PAD is shown in Figure 1.

**Figure 2:**
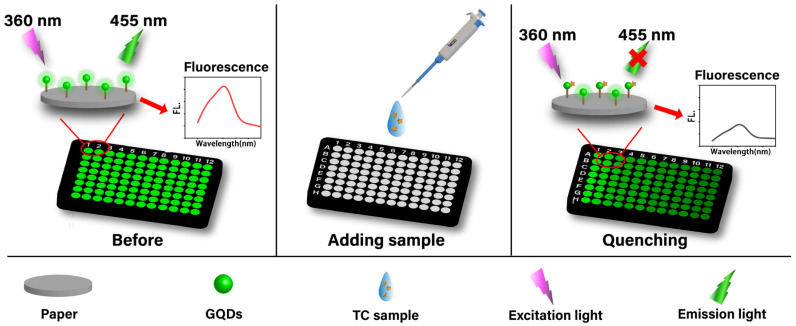
A schematic illustration of the detection process of the MIPs@GQDs@PAD for TC.

The sensing mechanism of this MIPs@GQDs@PAD for TC detection is discussed. This fluorescence quenching process may be caused by the internal filtration effect (IFE) and static quenching. The IFE occurs because the absorption bands of TC molecules range from 350 to 380 nm, which overlap with the absorption bands of GQDs. Therefore, the TC molecules absorb some of the photons used to excite the fluorescence of the GQDs, resulting in a decrease in the fluorescence intensity of the GQDs [6]. Additionally, another theory suggests that carboxyl groups on GQDs were negatively charged, while amino groups on TC were positively charged. This leads to a charge transfer between these groups, resulting in static quenching [7]. In static quenching, the quencher and the quantum dot form a stable ground-state complex, preventing the quantum dot from entering its excited state and thus reducing the overall fluorescence intensity. The sensing mechanism of using MIPs@GQDs@PAD is shown in Figure 2.

The quench effect can be fitted by using the Stern–Volmer equation:

(1) $$(F_{0}/F)-1=K_{SV}C_{M}$$

The Stern–Volmer equation assumes that the quenching process follows a dynamic mechanism, where $F_{0}$ represents the initial fluorescence intensity, and F is the fluorescence intensity in the presence of the quencher. *K_SV_* is the quenching constant, and *C_M_* is the concentration of the template molecules. The ratio of *K_SV_* values of MIPs and NIPs is defined as the imprinting factor (IF), a parameter used to characterize the selectivity of MIPs. A larger IF value indicates higher selectivity performance of MIPs@GQDs@PAD for target molecules.

### 3.2. Optical Characterization

**Figure 3:**
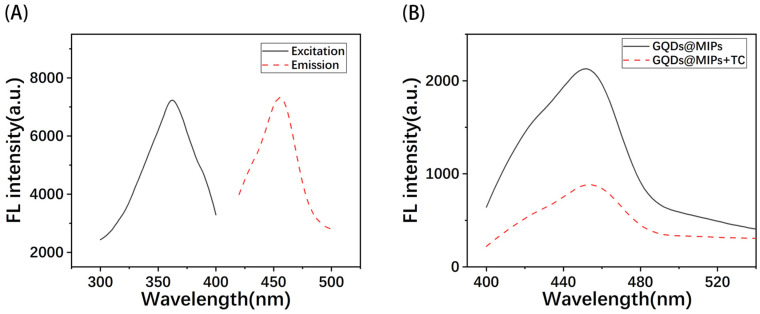
Fluorescence spectra of (**A**) MIPs@GQD emission spectra in the presence and absence of TC; (**B**) excitation and emission spectra of MIPs@GQDs@PAD.

To verify the fluorescence quenching effect of TC on MIPs@GQDs, the fluorescence emission spectra of MIPs@GQDs in the presence and absence of TC were determined, respectively. As shown in Figure 3A, the fluorescence intensity at 455 nm of the MIPs@GQD solution decreased significantly after TC was added. This result indicates that TC has a strong fluorescence quenching effect on MIPs@GQDs.

A full-wavelength scan was performed using a spectrophotometer to determine the optimal excitation wavelength (λex) and emission wavelength (λem) of MIPs@GQDs@PAD. The slit widths were set to 10 nm, and the scanning speed was 360 nm/min. The fluorescence spectrums in the λex range from 300 to 400 nm and in the λem range from 400 to 500 nm are shown in Figure 3B. It can be seen that the sensor exhibited the highest fluorescence intensity at an λex of 360 nm, and the fluorescence intensity of this sensor reaches its maximum at 455 nm. Moreover, the obtained fluorescence spectral curves are relatively smooth, indicating that the synthesized MIPs@GQD composites have good dispersion properties on the PAD. Therefore, the fluorescence intensity at 455 nm was used for detection by using λex at 360 nm in subsequent experiments.

**Figure 4:**
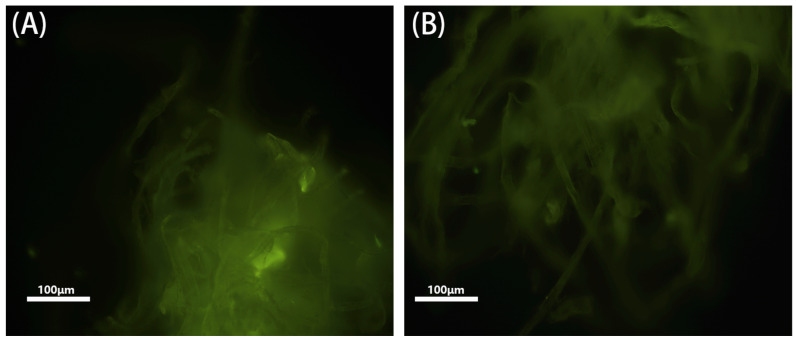
Fluorescence characterization of (**A**) MIPs@GQDs@PAD after elution and (**B**) MIPs@GQDs@PAD after readsorption of TC.

Fluorescence microscopy was employed to characterize the fluorescence images of MIPs@GQDs@PAD. Figure 4A shows that the image of eluted MIPs@GQDs@PAD exhibited a distinct fiber structure and uniform bright-green fluorescence. In contrast, Figure 4B displays significantly darker fluorescence, indicating quenching of the GQDs fluorescence after introducing TC molecules. These findings confirm the uniform distribution of MIPs@GQDs on the paper substrate and the quenching effect of TC molecules on the sensor’s fluorescence intensity.

### 3.3. SEM and FT-IR Characterization

**Figure 5:**
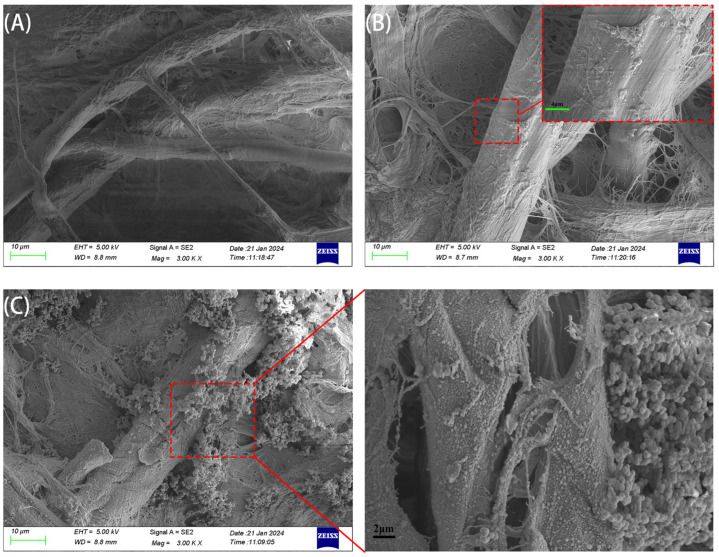
SEM characterization of (**A**) bare paper, (**B**) GQDs@PAD, and (**C**) MIPs@GQDs@PAD.

The micromorphologies of bare paper, GQDs@PAD, and MIPs@GQDs@PAD were characterized by scanning electron microscopy (SEM). As shown in Figure 5A, the bare paper exhibited an irregular dendritic cellulose structure with a relatively smooth surface. After treatment with APTES and grafting GQDs, obvious protrusions can be observed on the surface of paper fibers at high magnification (shown in Figure 5B), indicating that the GQDs were successfully grafted and uniformly distributed. Figure 5C shows that the paper surfaces were covered with many spherical polymer particles with diameters between 0.5 and 1 μm, illustrating that the MIPs were successfully synthesized on the PAD.

**Figure 6:**
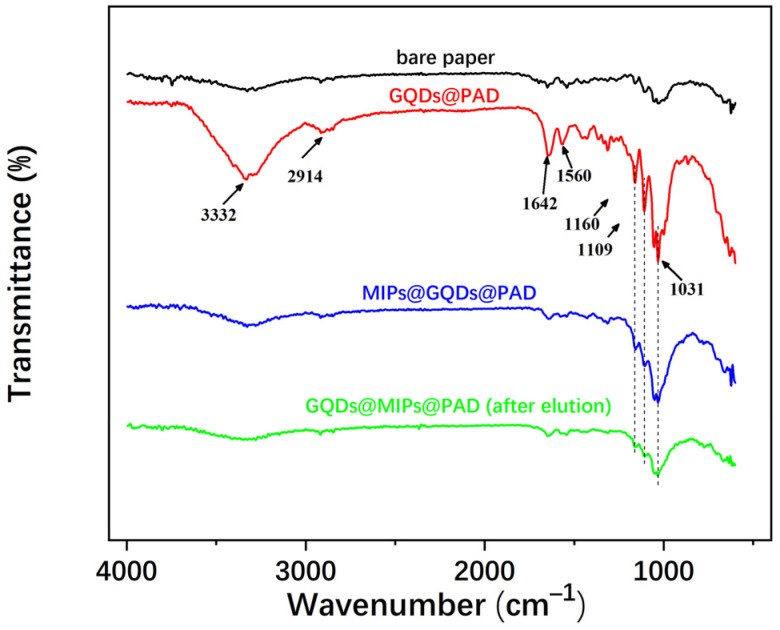
FT-IR spectra of bare paper, GQDs@PAD, and MIPs@GQDs@PAD before and after elution.

The chemical structures of bare paper, GQDs@PAD, MIPs@GQDs@PAD, and MIPs@GQDs@PAD after elution were characterized by Fourier-transform infrared spectroscopy (FT-IR) in ATR mode. As displayed in Figure 6, the absorption peak at 3332 cm^−1^ could be attributed to the N–H stretching vibration, and 1560 cm^−1^ is the N–H bending vibration, confirming that the -NH_2_ group of APTES was modified onto the paper surface [8]. The grafting of GQDs is demonstrated by the peak at 2914 and 1642 cm^−1^, which belongs to C–H and C=O stretching vibration [9]. Furthermore, the distinct characteristic peaks between 1031 and 1160 cm^−1^ correspond to Si-O and Si-O-Si. These peaks were weakened after the polymer layer coating. The above results indicated that the MIPs@GQDs@PAD has been successfully prepared.

### 3.4. Optimization of Experimental Conditions

The key parameters affecting the experiment were optimized to enhance the analytical performance of the MIPs@GQDs@PAD, such as the amount of GQDs, grafting time, the amount of functional monomer, pH value, eluent, and elution time.

#### 3.4.1. Effect of Grafting Time and Amount of GQDs

**Figure 7:**
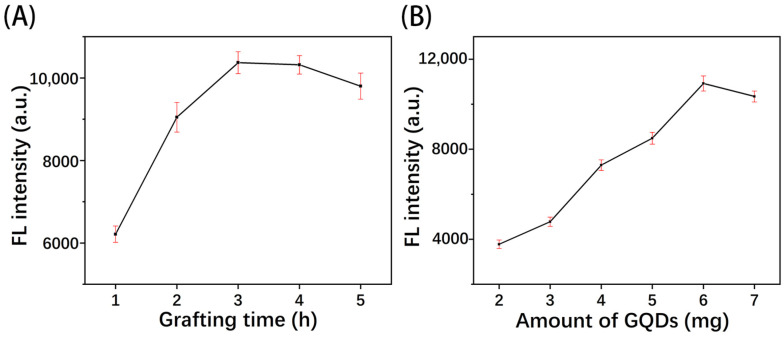
Effect of (**A**) GQD grafting time and (**B**) amount of GQDs.

The grafting time was optimized to ensure the bonding of GQDs and the paper substrate, enhancing sensor stability and batch consistency [10]. Figure 7A illustrates that when the grafting time was under 3 h, the fluorescence intensity increased with time, suggesting an incomplete reaction to form amide bonds. As time further increased, the fluorescence intensity gradually leveled off, indicating that the GQDs had been sufficiently bonded. Additionally, given that the acidic grafting solution can damage the paper fiber structure with prolonged processing, 3 h was determined to be the optimal grafting time.

The amount of GQDs was optimized as shown in Figure 7B. As the amount of GQDs increased within the range of 2~6 mg, the fluorescence intensity increased, reaching its maximum at 6 mg. The fluorescence signal is weak and easily saturated at low GQD concentrations, thus reducing the sensor’s sensitivity and detection range. However, when the amount of GQDs exceeds 6 mg, the fluorescence quenching due to GQDs’ agglomeration results in fluorescence intensity decreasing [11]. Therefore, 6 mg was chosen as the optimal amount of GQDs for subsequent experiments.

#### 3.4.2. Effect of pH

**Figure 8:**
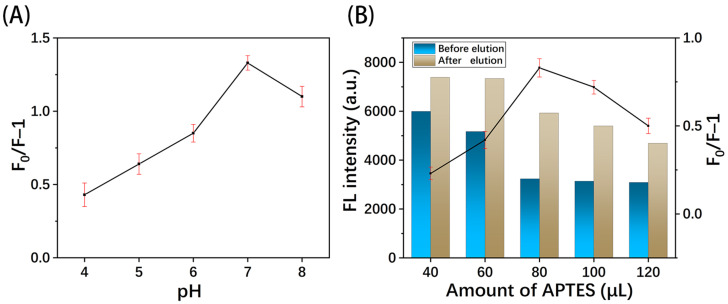
Effect of (**A**) pH and (**B**) amount of functional monomer.

The optimization of pH is shown in Figure 8A. When the pH was less than 6.0, the fluorescence quenching effect of TC was poor because the acidic environment reduces the emission of light by the GQDs [12]. As the pH increased, the fluorescence quenching effect was significantly enhanced and peaked at 7.0. When the solution was alkaline, the protonation of amino groups on the binding sites of MIPs weakened the interaction with TC molecules, thus reducing the quenching effect [12]. Considering the optimization results and practical applications in natural water bodies, the pH value of the detection environment in subsequent experiments was set at 7.0.

#### 3.4.3. Effect of Amount of Functional Monomer

The effect of the amount of functional monomer on MIP performance was investigated. As shown in Figure 8B, when 40 μL of APTES was used, there was little difference in fluorescence intensity before and after elution (F_0_/F). This is due to the low crosslinking polymer’s inability to form effective recognition sites, resulting in poor adsorption of TC molecules in the MIPs [13]. As the APTES further increased to 120 μL, the fluorescence intensity remained almost unchanged, while the F_0_/F was relatively decreased. This is attributed to the excess functional monomers forming highly rigid recognition sites, increasing the difficulty of eluting the template molecules. Thus, 80 μL was chosen as the optimal amount of functional monomer.

#### 3.4.4. Effect of Eluent and Elution Times

**Figure 9:**
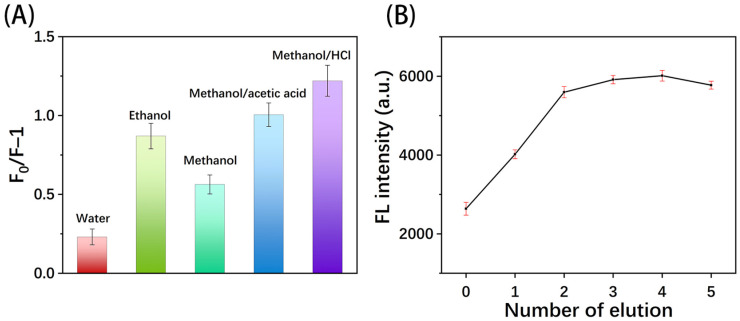
Effect of (**A**) different eluents and (**B**) number of elution.

The correct eluent and appropriate elution time can ensure the effective removal of templates from MIPs. Five eluents were selected for testing, and all the eluents were performed twice for 5 min each. The results are shown in Figure 9A, indicating that the highest F_0_/F value was obtained by using methanol/hydrochloric acid (0.5 mol/L, 9:1, *v*/*v*). Figure 9B shows the optimization of the elution times, where the fluorescence intensity recovered rapidly as the elution time increased. The fluorescence intensity tends to be stable after the second elution, indicating that most of the template molecules have been removed from the MIPs. Since the acidic eluent adversely affected the paper fiber structure, elution was chosen twice for subsequent experiments.

### 3.5. Analytical Performance of MIPs@GQDs@PAD

**Figure 10:**
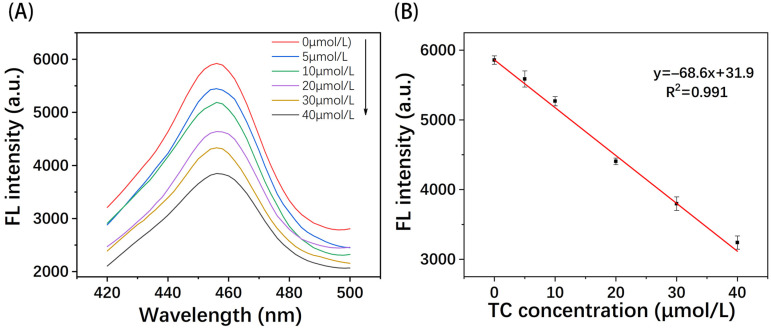
The (**A**) fluorescence emission spectra of MIPs@GQDs@PAD under different TC concentrations and the (**B**) calibration curve and linear equation.

The analytical performance of MIPs@GQDs@PAD for TC detection was investigated under optimized experimental conditions. As illustrated in Figure 10A, the fluorescence intensity decreased with the increase in TC concentration and showed a good linear relationship in the 0–40 µmol/L range. The calibration curve is shown in Figure 10B, which is described by the linear equation y = −68.6x + 31.9 with a correlation coefficient (R^2^) = 0.991. The limit of detection (LOD) of 0.87 µmol/L was calculated from 3σ/S, where σ is the standard deviation of the blank sample, and S is the slope of the calibration curve also defined as the sensor’s sensitivity (68.6 µmol/L/AU).

**Figure 11:**
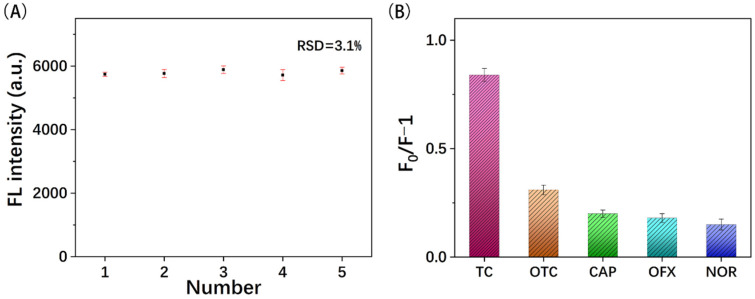
The (**A**) reproducibility and (**B**) selectivity of MIPs@GQDs@PAD.

The reproducibility and selectivity of MIPs@GQDs@PAD were tested before analyzing actual samples. Five sensors were prepared under parallel experimental conditions, and their fluorescence intensities were detected. As shown in Figure 11A, the RSD was 3.1%, demonstrating that the sensor had good reproducibility. To evaluate the specific recognition ability of MIPs@GQDs@PAD for TC, antibiotic analogs with similar structures to TC, such as oxytetracycline (OTC) and chloramphenicol (CAP), were chosen as interferents for the selectivity experiments. Figure 11B demonstrates that TC exhibits more excellent fluorescence quenching than the interferents. This is because the imprinting sites of MIPs, which complement the shape and functional groups of TC molecules, facilitate stronger binding with TC. The selectivity experiment results showed that MIPs@GQDs@PAD had a better ability to recognize TC, and common analogs specifically did not cause any significant interference with the test results. In addition, Table 1 indicates the performance of the MIPs@GQDs@PAD compared to other reported fluorescence sensors for TC detection.

### 3.6. Application of MIPs@GQDs@PAD for TC Detection in Real Samples

To validate the practicality of MIPs@GQDs@PAD for detecting TC, river and tap water samples were obtained around Beijing City. All water samples were pretreated with a 0.22 µm filter membrane, and the pH was adjusted to 7.0. TC was added to the water samples to prepare spiked solutions with concentrations of 5 µmol/L and 10 µmol/L, respectively. Table 2 shows recoveries in river water and tap water samples ranging from 103.3% to 109.4%, with relative standard deviations (RSDs) ranging from 4.4% to 6.4%. The results of actual samples show that MIPs@GQDs@PAD demonstrates acceptable accuracy for the detection of TC and can be used for accurate sample analysis.

## 4. Conclusions

In this work, we developed a novel, portable paper-based fluorescent sensor for the rapid quantitative detection of tetracycline. To the best of our knowledge, this is the first time researchers have realized the detection of tetracycline on a paper-based platform using graphene quantum dots coated with molecularly imprinted polymer. MIPs@GQDs were synthesized on a solid-phase paper substrate by chemically grafting GQDs and through the precipitation polymerization of MIPs, thus improving sensor portability. Compared to metal QDs, GQDs are non-toxic, eco-friendly, and have better biocompatibility. Unlike other fluorescent PADs, our proposed sensor is compatible with a microplate reader, enabling the multi-channel detection of multiple samples. The method offers various attractive benefits, including speed, portability, low costs, and being user-friendly. The method exhibited good reproducibility and selectivity when detecting TC in actual water samples. It is expected that the proposed MIPs@GQDs@PAD has excellent potential for application in environmental point-of-care testing of antibiotics.

## Figures and Tables

**Table 1:** Comparison of MIPs@GQDs@PAD performance with other reported fluorescence sensors for TC detection.

Fluorescence Sensors	Linear Ranges (µmol/L)	Limit of Detections (µmol/L)	Refs.
MIP–g/r-QD	10–160	0.35	[14]
MIPs-AA/CQDs	1–60	0.17	[15]
FeO_x_@SiO_2_-FMIPs	0.2–6	0.117	[16]
MIPs-ZnO NRs	5–120	1.02	[17]
MIPs@GQDs@PAD	0–40	0.87	This work

**Table 2:** Recovery of detecting TC in real water samples using MIPs@GQDs@PAD.

Sample	Add (μmol/L)	Found (μmol/L)	Recovery (%)	RSD (%, n = 3)
Tap water	0	-	-	-
	5	5.47	109.4%	5.3%
	10	10.81	108.1%	4.8%
River water	0	-	-	-
	5	5.36	107.2%	6.4%
	10	10.33	103.3%	4.4%

## Data Availability

The original contributions presented in the study are included in the article, further inquiries can be directed to the corresponding authors.
